# Genome-wide 5-hydroxymethylcytosine patterns in human spermatogenesis are associated with semen quality

**DOI:** 10.18632/oncotarget.18331

**Published:** 2017-06-01

**Authors:** Olga A. Efimova, Anna A. Pendina, Andrei V. Tikhonov, Sergey E. Parfenyev, Irina D. Mekina, Evgeniia M. Komarova, Mariia A. Mazilina, Eugene V. Daev, Olga G. Chiryaeva, Ilona A. Galembo, Mikhail I. Krapivin, Oleg S. Glotov, Irina S. Stepanova, Svetlana A. Shlykova, Igor Yu. Kogan, Alexander M. Gzgzyan, Tatyana V. Kuznetzova, Vladislav S. Baranov

**Affiliations:** ^1^ D.O. Ott Research Institute of Obstetrics, Gynecology and Reproductology, St. Petersburg, Russia; ^2^ St. Petersburg State University, St. Petersburg, Russia; ^3^ Center for Medical Genetics, St. Petersburg, Russia; ^4^ St. Petersburg State Pediatric Medical University, St. Petersburg, Russia; ^5^ S.M. Kirov Military Medical Academy, St. Petersburg, Russia; ^6^ Institute of Cytology RAS, St. Petersburg, Russia; ^7^ Aimed Clinic, St. Petersburg, Russia; ^8^ AVA-Peter Clinic, St. Petersburg, Russia

**Keywords:** 5-hydroxymethylcytosine, semen quality, sperm DNA fragmentation, human spermatogenesis, testicular spermatogenic cells, Pathology Section

## Abstract

We performed immunofluorescent analysis of DNA hydroxymethylation and methylation in human testicular spermatogenic cells from azoospermic patients and ejaculated spermatozoa from sperm donors and patients from infertile couples. In contrast to methylation which was present throughout spermatogenesis, hydroxymethylation was either high or almost undetectable in both spermatogenic cells and ejaculated spermatozoa. On testicular cytogenetic preparations, 5-hydroxymethylcytosine was undetectable in mitotic and meiotic chromosomes, and was present exclusively in interphase spermatogonia Ad and in a minor spermatid population. The proportions of hydroxymethylated and non-hydroxymethylated diploid and haploid nuclei were similar among samples, suggesting that the observed alterations of 5-hydroxymethylcytosine patterns in differentiating spermatogenic cells are programmed. In ejaculates, a few spermatozoa had high 5-hydroxymethylcytosine level, while in the other ones hydroxymethylation was almost undetectable. The percentage of highly hydroxymethylated (5-hydroxymethylcytosine-positive) spermatozoa varied strongly among individuals. In patients from infertile couples, it was higher than in sperm donors (*P*<0.0001) and varied in a wider range: 0.12-21.24% versus 0.02-0.46%. The percentage of highly hydroxymethylated spermatozoa correlated strongly negatively with the indicators of good semen quality – normal morphology (*r*=-0.567, *P*<0.0001) and normal head morphology (*r*=-0.609, *P*<0.0001) – and strongly positively with the indicator of poor semen quality: sperm DNA fragmentation (*r*=0.46, *P*=0.001). Thus, the immunocytochemically detected increase of 5hmC in individual spermatozoa is associated with infertility in a couple and with deterioration of sperm parameters. We hypothesize that this increase is not programmed, but represents an induced abnormality and, therefore, it can be potentially used as a novel indicator of semen quality.

## INTRODUCTION

Human spermatozoa have a unique epigenome which is formed through epigenetic changes during a long period of differentiation from primordial germ cells to mature gametes. The establishment of appropriate epigenetic patterns through DNA demethylation/remethylation, chromatin remodeling and non-coding RNAs is integral to achieving high spermatozoon specialization, which is required for the transmission of genetic and epigenetic information and initiation of embryo development [1].

A major mechanism of human genome reprogramming is DNA methylation [2-4]. Establishment, maintenance and alteration of specific 5-methylcytosine (5mC) patterns are the key aspects in cell fate determination through arrangement of DNA-protein interactions and activation / repression of specific gene expression programs [5]. Errors in these processes are associated with various human pathologies [6-10]. Along with 5mC, the DNA methylation/demethylation cycle includes three other forms of modified cytosine: 5-hydroxymethylcytosine (5hmC), 5-formylcytosine and 5-carboxylcytosine. They are produced by ten eleven translocation (TET)-mediated 5mC oxidation as intermediate products in active DNA demethylation [11, 12]. However, many current studies discuss the epigenetic effect of 5mC oxidative derivatives, especially of 5hmC, in genome function regulation [13-15].

DNA methylation patterns in mature spermatozoa are rather well studied [16-18]. The sperm methylome differs markedly from that of somatic cells, but is similar to that in embryonic stem cells and embryonic germ cells [16]. The establishment of germ-cell-specific DNA methylation signatures is a highly determined process. However, the reversibility of cytosine modification mechanisms provides methylome plasticity, which is believed to be important for adaptation to environmental factors through alterations of genome activity [19, 20]. In human, the development of germ cells from spermatogonia to spermatozoa takes 74 days [21, 22] and is accompanied by a decrease in the amount of cytoplasm which makes both genetic and epigenetic patterns in spermatogenic cells and mature spermatozoa highly vulnerable to internal and external factors [23]. Aberrant DNA methylation changes in sperm have been shown to increase risk of reproductive failures [24], and to deregulate gene expression and promote genome instability [25, 26]. Moreover, specific alterations of germline DNA methylation are associated with variation in sperm morphology and motility [27-29].

Considering that DNA methylation may be changed through 5mC hydroxylation and that the 5hmC itself has a regulatory role in gene expression, it is of both fundamental and practical importance to study DNA hydroxymethylation patterns in human spermatogenesis. To date, sperm hydroxymethylation patterns have been reported only in few studies, which have quantified 5hmC in DNA from multi-cell samples: absolute level in the whole ejaculate [30, 31] and gene-specific hydroxymethylation levels in the populations of normal, abnormal and globozoospermic spermatozoa [32]. However, the analysis of genomic DNA extracted from multi-cell samples shows an average hydroxymethylation level leaving the possibility that a simultaneous increase of 5hmC level in some cells and a decrease in others remains undetected.

In this study, we analyze genome-wide 5hmC and 5mC patterns in human testicular spermatogenic cells and ejaculated spermatozoa, using immunocytochemical detection *in situ*. This technique allows single-cell analysis of large cell populations in each individual and, importantly, allows revealing the discreteness of germ cell epigenetic marking. We address the following questions concerning 5hmC patterns in normal and pathological human spermatogenesis: 1) whether sperm 5hmC patterns show inter-cell and inter-individual variation in sperm donors versus patients from infertile couples; 2) whether sperm 5hmC patterns are associated with semen parameters; and 3) whether 5hmC patterns undergo global changes during differentiation of spermatogenic cells.

## RESULTS

### Sperm 5-hydroxymethylcytosine and 5-methylcytosine patterns

**Figure 1:**
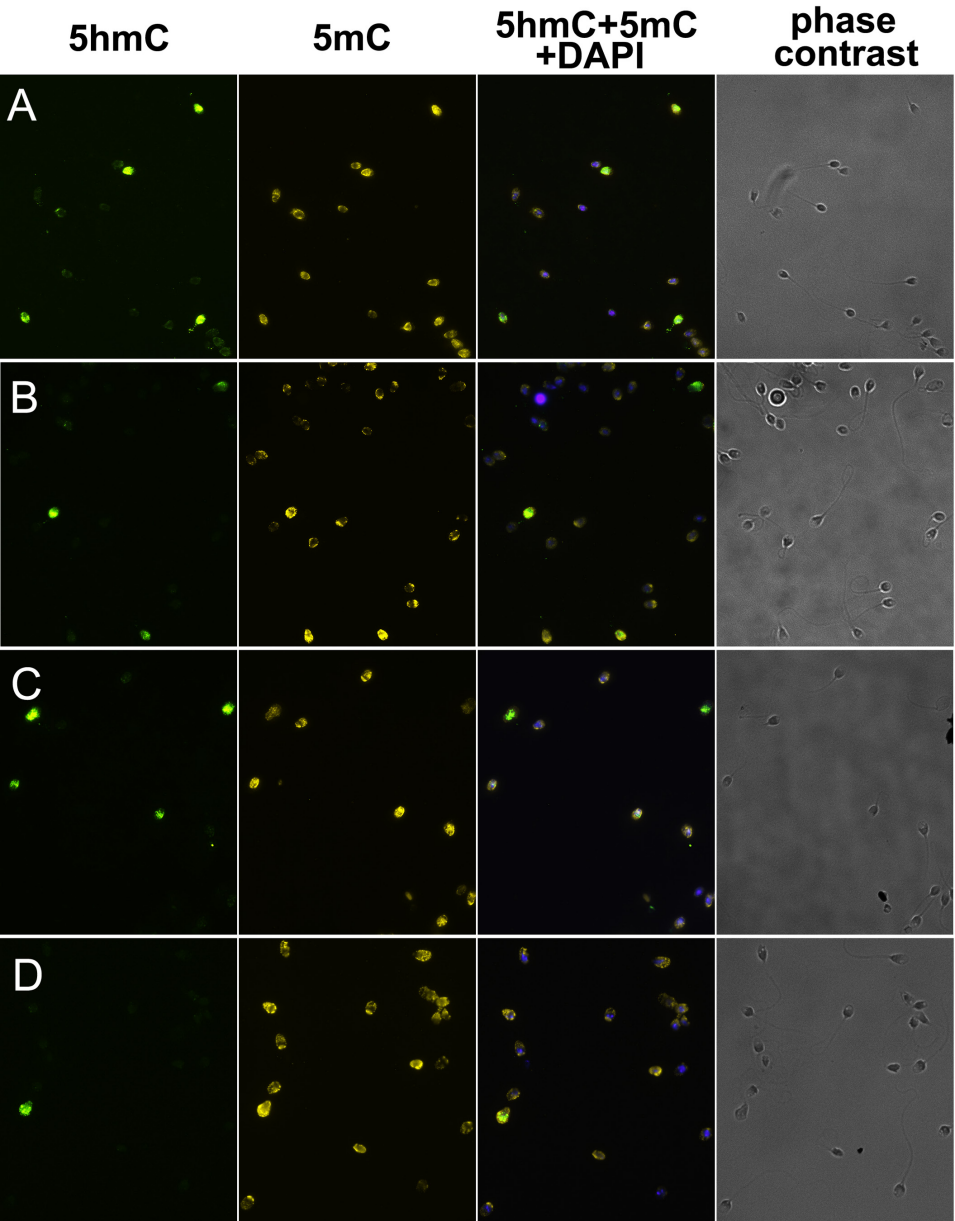
5-hydroxymethylcytosine (5hmC) and 5-methylcytosine (5mC) patterns in human ejaculated spermatozoa Immunostaining for 5hmC, 5mC, merge image, and phase-contrast images of spermatozoa from patients (**A**-**C)** and a sperm donor (**D),** having 10.04 %, 6.04 %, 21.24 %, and 0.46 % of 5hmC-positive spermatozoa in ejaculate, respectively.

**Figure 2:**
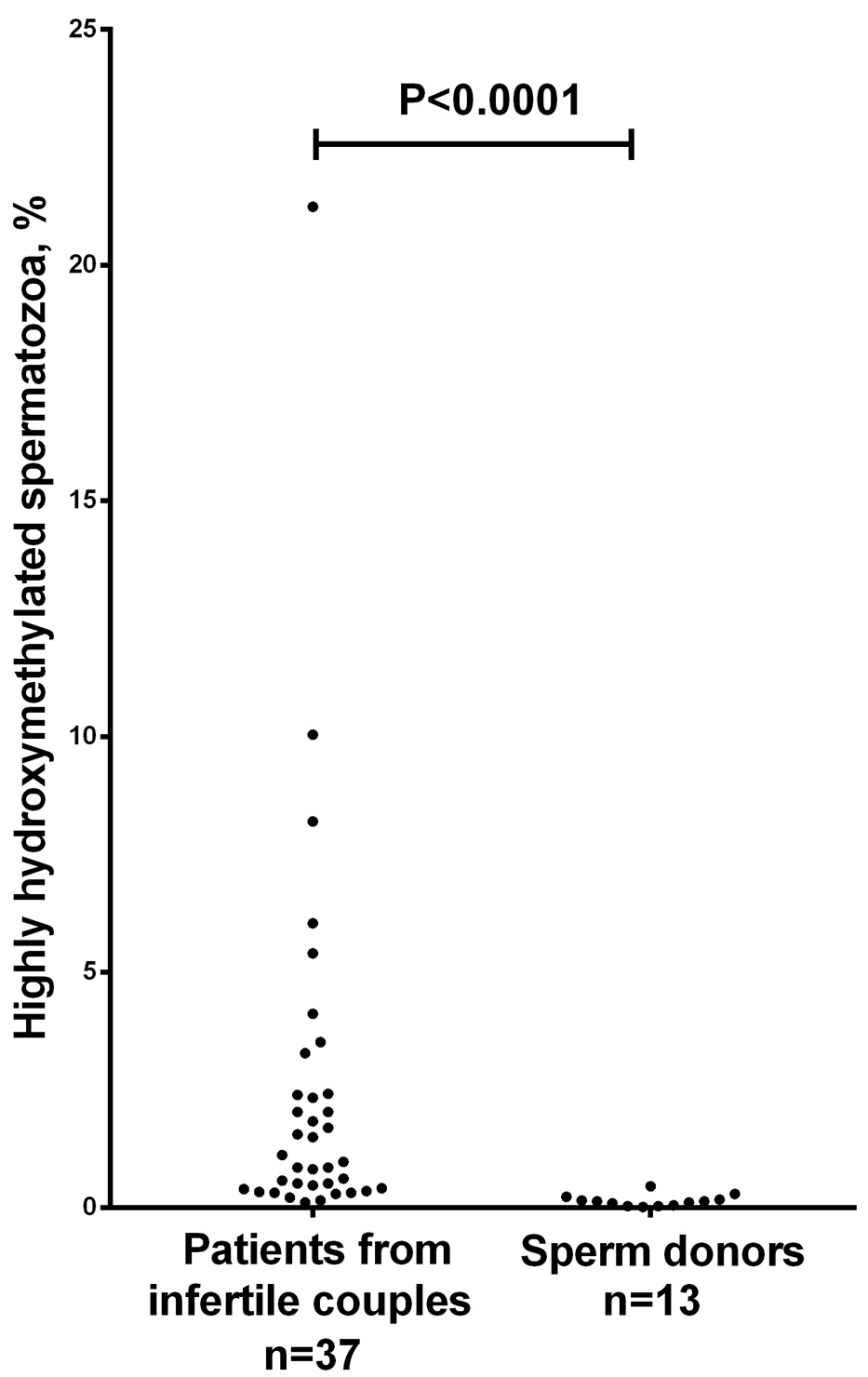
Scatter dot plot showing the percentage of immunocytochemically detected highly hydroxymethylated (5hmC-positive) spermatozoa in ejaculates from sperm donors versus patients from infertile couples *The frequency of 5hmC-positive spermatozoa is significantly higher in patients compared to sperm donors (*P* < 0.0001, the Mann-Whitney U-test). All 5hmC-positive spermatozoa had visible anti-5-methylcytosine fluorescence.

Using the immunofluorescent approach, we evaluated the presence of 5hmC and 5mC in human ejaculated spermatozoa fixed on glass slides. Inter-cellular variability in anti-5hmC fluorescence intensity was strongly pronounced: 5hmC was almost completely absent in most spermatozoa and only a few of them had high hydroxymethylation level (Figure 1). The frequency of highly hydroxymethylated (5hmC-positive) spermatozoa in the patients from infertile couples was significantly higher than that in the sperm donors: 0.12-21.24 % versus 0.02-0.46 % (*P* < 0.0001) (Figure 2). The range of deviation in patients was 48 fold higher than in sperm donors.

**Figure 3:**
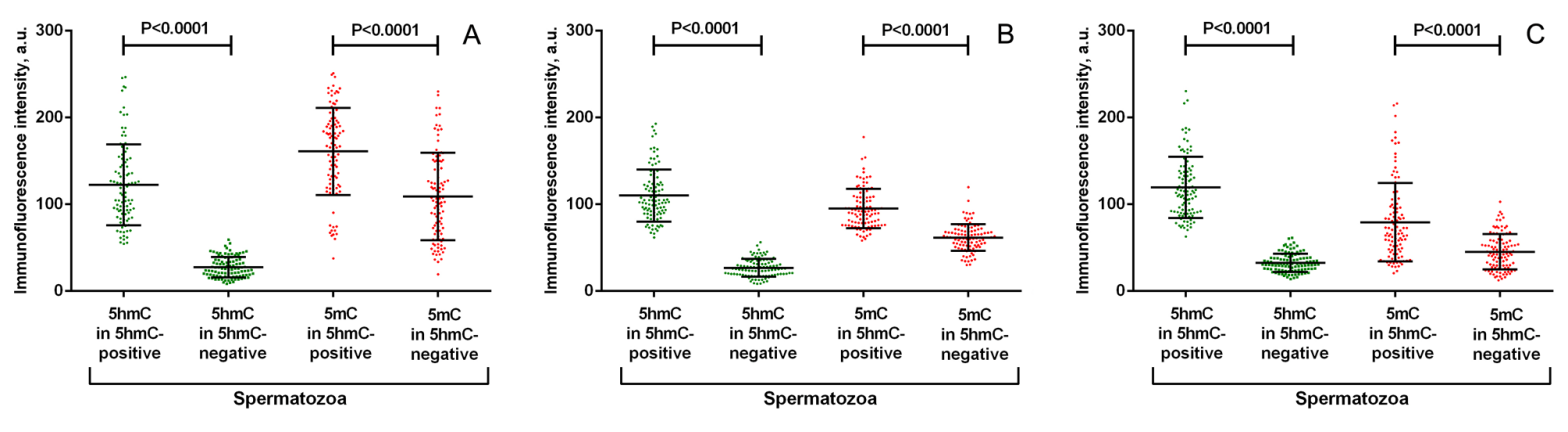
Quantitative analysis of anti-5-hydroxymethylcytosine (5hmC) and anti-5-methylcytosine (5mC) fluorescence intensity in representative spermatozoa from 3 individuals (A, B, C) * Both anti-5hmC and anti-5mC fluorescence intensities differ significantly between 5hmC-positive and 5-hmC-negative spermatozoa (*P* < 0.0001, the Mann-Whitney U-test).

In contrast to 5hmC, 5mC was present in all spermatozoa to an easily detectable level (Figure 1). Inter-cellular variability in anti-5mC fluorescence intensity was not obviously visible by the qualitative analysis. However, the quantitative analysis of anti-5mC fluorescence intensity showed a higher methylation level in 5hmC-positive spermatozoa (*P* < 0.0001) (Figure 3).

### Sperm 5-hydroxymethylcytosine pattern in relation to semen parameters

Using correlation analysis, we checked the strength and the direction of relationship between the highly hydroxymethylated spermatozoa percentage and other sperm parameters in 50 individuals analyzed in this study. The sperm parameters included sperm DNA integrity, assessed by terminal deoxynucleotidyl transferase-mediated dUTP nick-end labelling (TUNEL) on glass slides, and standard quantitative and qualitative sperm characteristics: concentration, motility and morphology, including normal head, abnormal head, abnormal tail, and abnormal midpiece.

**Figure 4:**
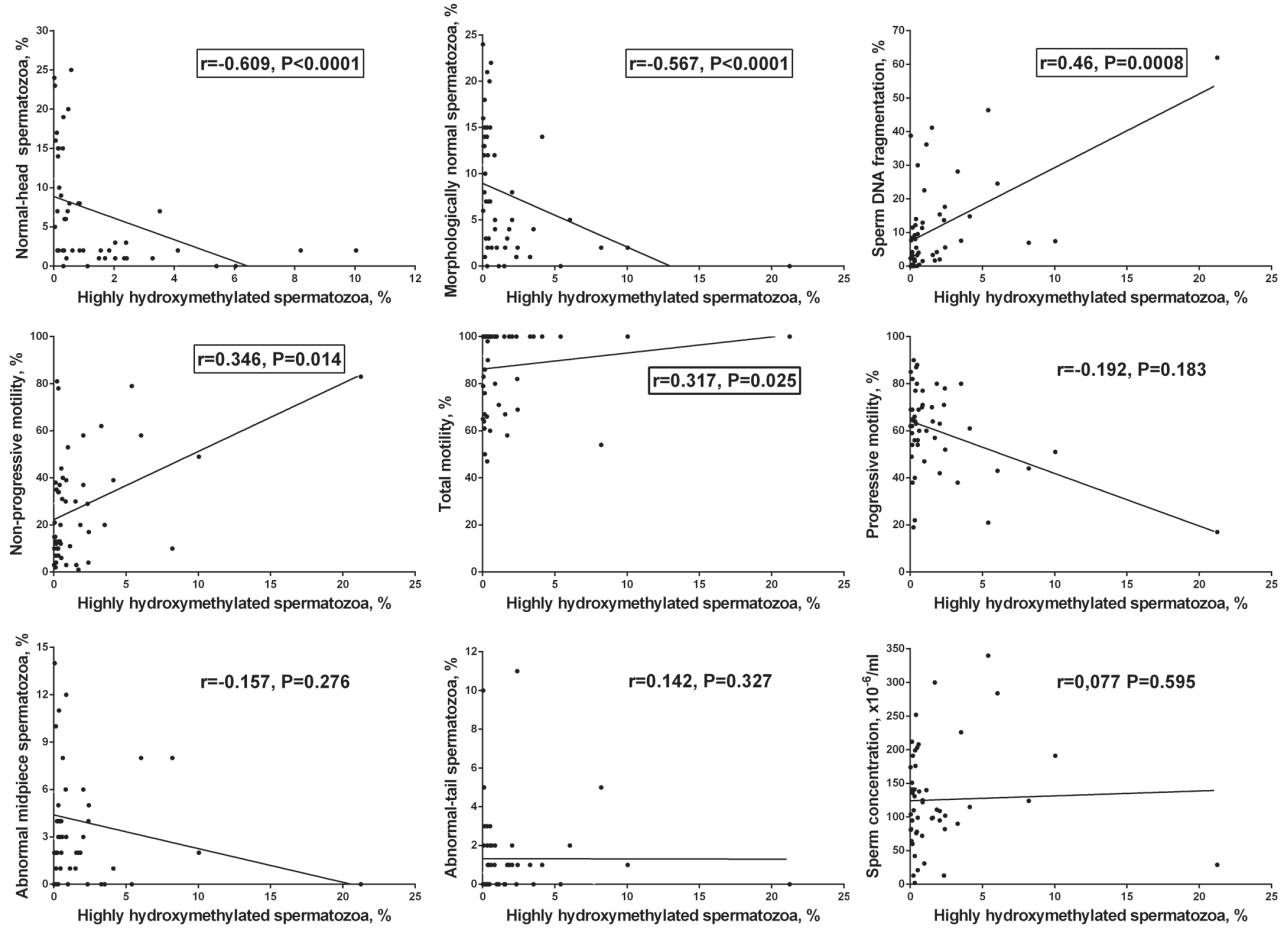
Correlations between the frequency of highly hydroxymethylated (5hmC-positive) spermatozoa in human ejaculate and semen parameters Statistically significant correlations are framed (*P* < 0.05, the nonparametric Spearman test).

The percentage of highly hydroxymethylated spermatozoa showed a strong positive correlation with the frequency of TUNEL-positive spermatozoa (*r* = 0.46, *P* = 0.001) and a strong negative correlation with the frequency of morphologically normal (*r* = -0.567, *P* < 0.0001) and normal-head spermatozoa (*r* = -0.609, *P* < 0.0001). A weak positive correlation was observed with non-progressive motility (*r* = 0.346, *P* = 0.014) and with total motility (*r* = 0.317, *P* = 0.025). No statistically significant correlations were found between the percentage of highly hydroxymethylated spermatozoa and other parameters, including concentration (*r* = 0.077, *P* = 0.595), progressive motility (*r* = -0.192, *P* = 0.183), abnormal-tail spermatozoa (*r* = 0.142, *P* = 0.327), or abnormal midpiece (*r* = -0.157, *P* = 0.276) (Figure 4).

Thus, the frequency of highly hydroxymethylated spermatozoa in ejaculate is associated with a variation in sperm parameters, showing strong negative correlation with indicators of good semen quality - the frequency of morphologically normal and normal-head spermatozoa, and strong positive correlation with the indicator of poor semen quality - the frequency of TUNEL-positive spermatozoa.

### DNA hydroxymethylation and methylation patterns in spermatogenic cells

We have studied DNA hydroxymethylation and methylation patterns in human spermatogenic cells from testicular tissue samples of azoospermic patients. We performed simultaneous immunodetection of 5hmC and 5mC on testicular cytogenetic preparations - chromosomes and nuclei fixed on glass slides and stained with quinacrine-fluorescence-Hoechst (QFH). We identified spermatogonial mitotic chromosomes at metaphase stage and spermatocyte meiotic chromosomes at prophase I (pachytene, diplotene, and diakinesis) and metaphase II stages.

**Figure 5:**
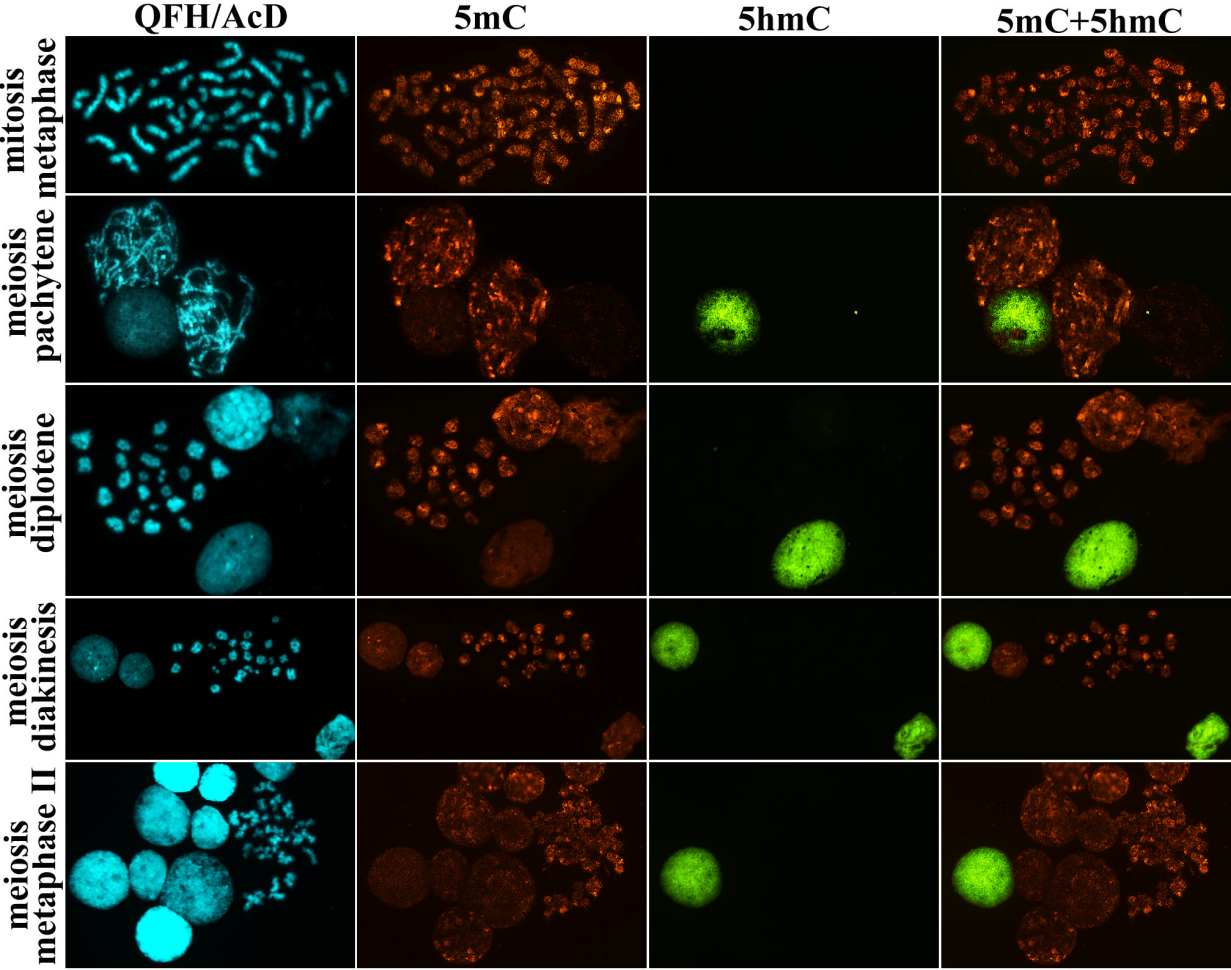
5-hydroxymethylcytosine (5hmC) and 5-methylcytosine (5mC) patterns in human testicular cells: mitotic spermatogonium at metaphase stage, meiotic spermatocytes at prophase (pachytene, diplotene and diakinesis) stage and metaphase II stage after QFH/AcD banding and immunodetection of 5hmC and 5mC In both mitotic and meiotic chromosomes from spermatogenic cells 5hmC is undetectable, whereas 5mC is present. QFH/AcD staining and photoimaging of banded chromosomes were performed before the 5hmC and 5mC immunodetection.

When analyzing DNA methylation pattern, we detected 5mC in all nuclei and in both mitotic and meiotic chromosomes (Figure 5). There was no obvious inter-nuclei difference in DNA methylation intensity. The distribution of 5mC along the arms of spermatogonial mitotic chromosomes was band-specific. The 5mC-richest chromosome regions corresponded to QFH-negative euchromatic bands (R-bands), whereas QFH-positive euchromatic bands (G-bands) were methylated to a lesser extent (Figure 5).

A specific 5mC distribution pattern was also detected in the meiotic chromosomes. In the pachytene chromosomes, 5mC was present in interchromomeric segments with a higher methylation level in near-telomeric regions. Similarly, in the diplotene, diakinetic, and metaphase II chromosomes, methylation was present non-randomly along arms (Figure 5).

**Figure 6:**
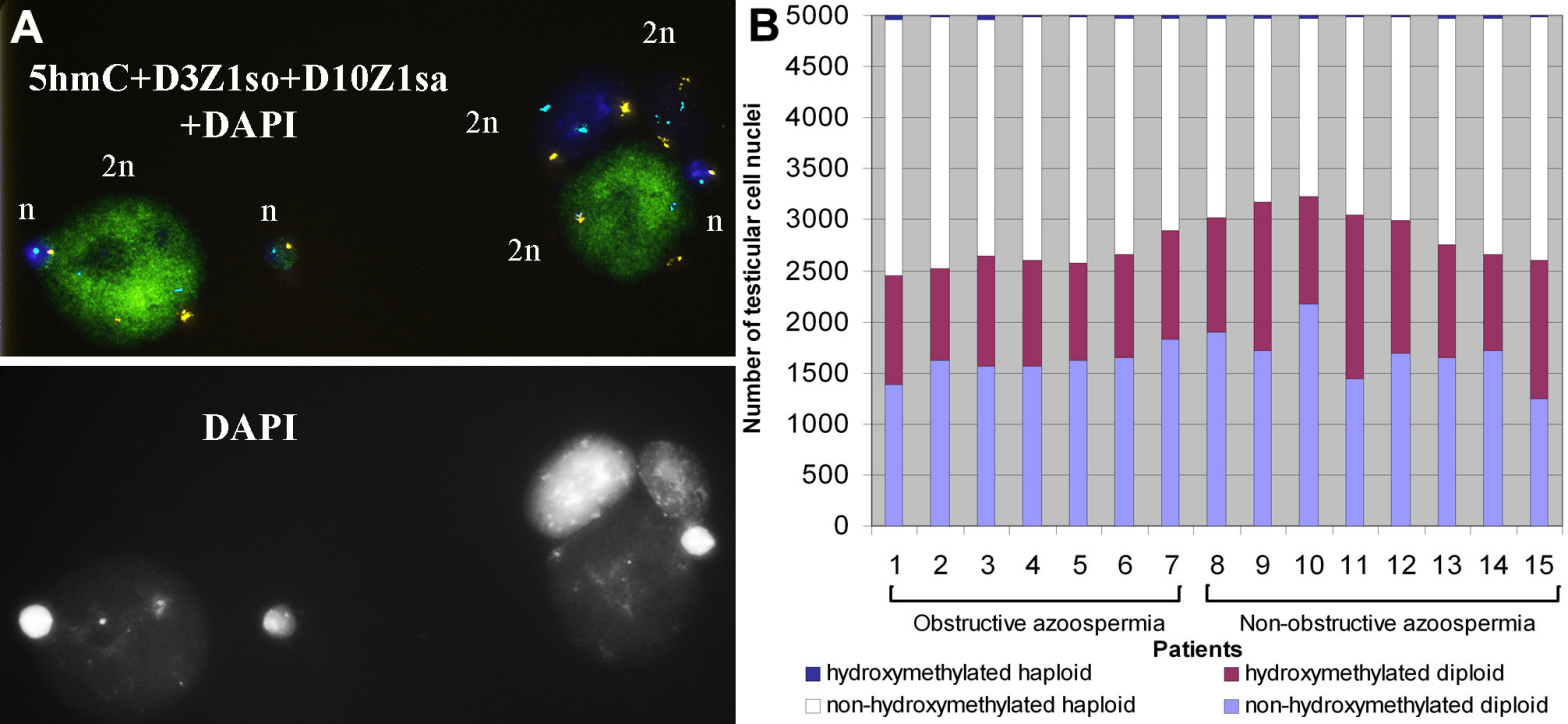
5-hydroxymethylcytosine (5hmC) patterns in nuclei of human testicular cells **A.** The haploid and diploid nuclei of testicular cells after subsequent fluorescence in situ hybridization (FISH) with DNA probes specific for centromere 3 (3p11.1-q11.1 = D3Z1, SpectrumOrange) and 10 (10p11.1-q11.1 = D10Z1, SpectrumAqua) and immunostaining for 5hmC/Alexa488 (green) with DAPI counterstaining. In the shown field of view, two out of four diploid nuclei and one out of three haploid nuclei are hydroxymethylated, whereas in the rest nuclei 5hmC is undetectable. **B.** The proportions of non-hydroxymethylated diploid, hydroxymethylated diploid, non-hydroxymethylated haploid and hydroxymethylated haploid nuclei among a total of 5000 nuclei in each of testicular tissue samples from 15 azoospermic patients. The proportions of nucleus types do not differ between patients with obstructive and non-obstructive azoospermia (P = 0.978, Fisher’s z-test).

When examining 5hmC distribution in the chromosomes and nuclei on the same preparations, we revealed a remarkably different pattern from that of 5mC. 5hmC level was extremely low/undetectable in both mitotic and meiotic chromosomes, including prophase I (pachytene, diplotene, and diakinesis) and metaphase II stages (Figure 5). Hydroxymethylation in interphase nuclei was present either at high or at extremely low/undetectable level (Figure 6A).

We determined whether the nuclei were from premeiotic/somatic or postmeiotic cells based on their ploidy, revealed by fluorescence in situ hybridization (FISH) prior to the immunodetection of 5hmC. Four types of nuclei were detected: hydroxymethylated diploid, non-hydroxymethylated diploid, hydroxymethylated haploid, and non-hydroxymethylated haploid nuclei (Figure 6A). The proportions of nucleus types did not differ between patients with obstructive and non-obstructive azoospermia (*P* = 0.978), suggesting that stages of spermatogenesis were similarly represented in all the analyzed individuals (Figure 6B). The percentage of hydroxymethylated nuclei among diploid ones varied from 32.62 to 52.57 % in different samples (mean 40.75±5.89). Among hydroxymethylated diploid nuclei, we observed the prevalence of nuclei with a large vacuole - a specific characteristic of Ad, but not Ap or B spermatogonia [33]. The diploid hydroxymethylated nuclei with no vacuole, which might be either from somatic or from spermatogenic cells, amounted to no more than 1-2 %. The percentage of hydroxymethylated nuclei among haploid ones varied from 0.42 to 1.96 % in different samples (mean 1.18±0.51).

On the testicular cytogenetic preparations from 13 out of 15 patients, we detected a total of 283 spermatozoa with no highly hydroxymethylated ones. All the analyzed spermatozoa had basically low 5hmC level.

**Figure 7:**
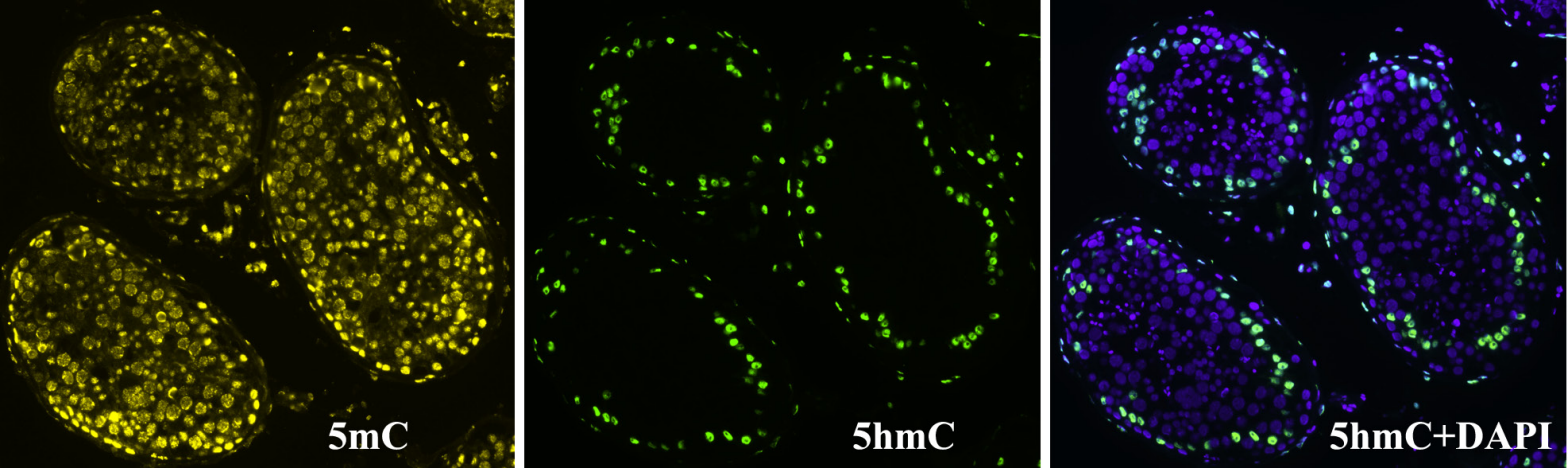
Immunohistochemical detection of 5-methylcytosine (5mC) and 5-hydroxymethylcytosine (5hmC) patterns in a tubular cross section 5mC is visible in nuclei of all cell types within the tubules, while 5hmC is present mostly in spermatogonia Ad lying next to the basal membrane.

We then performed immunohistochemical staining of testis tissue sections detecting 5hmC and 5mC. In accordance with our results on cytogenetic preparations, 5mC was present in all cell types within the tubules, while 5hmC was visible only in some cells. The hydroxymethylated cells mostly located near the basal membrane and had vacuoles (Figure 7). Thus, in contrast to 5mC, which is present in all testicular cells, 5hmC locates almost exclusively in nuclei of spermatogonia Ad and in nuclei of only a few spermatids.

### 5-hydroxymethylcytosine and 5-methylcytosine patterns in unfertilized oocytes

**Figure 8:**
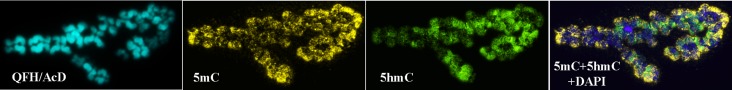
Human meiotic oocyte after QFH/AcD staining and immunodetection of 5-methylcytosine (5mC) and 5-hydroxymethylcytosine (5hmC) In contrast to mitotic and meiotic chromosomes from spermatogenic cells, oocyte chromosomes are methylated and hydroxymethylated along arms. QFH/AcD staining and photoimaging of banded chromosomes were performed before the 5hmC and 5mC immunodetection.

We have analyzed 5hmC and 5mC patterns in unfertilized human oocytes. Unfertilized oocytes were fixed on glass slides and meiotic chromosomes at metaphase II stage were available for analysis. In contrast to meiotic spermatocytes and most mature spermatozoa, 5mC and 5hmC levels in oocyte chromosomes were comparable. Chromosomes were densely methylated and hydroxymethylated along arms with the only exception of pericentric regions, which contained neither 5mC nor 5hmC (Figure 8). This pattern contrasted sharply to that in spermatocyte metaphase II chromosomes, which featured methylation along arms, including pericentric regions, and absence of hydroxymethylation (Figure 5).

## DISCUSSION

The role of epigenetics in human reproduction is being intensively studied. Recent studies have provided evidence that aberrantly changed 5mC patterns in sperm are associated with male infertility [27-29]. In the present study, we focus on 5hmC - an oxidative derivative of 5mC which can appear during programmed genome epigenetic changes or in response to endo- and exogenous factors. We report immunofluorescently detected 5hmC patterns in human ejaculated spermatozoa and in testicular spermatogenic cells, considering their DNA methylation status. We show that 5mC is present throughout studied stages of spermatogenesis. In contrast to 5mC, 5hmC is present either at high or at extremely low / undetectable level in both ejaculated spermatozoa and testicular spermatogenic cells (Figure 1, 5, 6A). The presence of high 5hmC level in some, but not all germ cells, raises a question about the reasons for such pattern.

Based on our findings, we suggest that a higher frequency of 5hmC-positive spermatozoa indicates poor semen quality. Firstly, an increase in highly hydroxymethylated spermatozoa frequency in ejaculate is more typical for patients from infertile couples than for healthy sperm donors. Secondly, the frequency of 5hmC-positive spermatozoa correlates strongly negatively with some indicators of good semen quality (morphologically normal spermatozoa and normal-head spermatozoa) and strongly positively with sperm DNA fragmentation, which is an indicator of poor semen quality. Thus, an increase in frequency of highly hydroxymethylated spermatozoa may be associated with fertility impairment.

An essential question is by what mechanism spermatozoa acquire 5hmC. There are two pathways of 5hmC formation - by programmed TET-mediated 5mC oxidation [11] and by a random hydroxyl radical attack on 5mC under oxidative stress conditions [34, 35]. In ejaculated spermatozoa, *TET* mRNA levels are significantly reduced in subfertile patients [36], indicating the possibility that a high sperm hydroxymethylation may be spontaneously induced by reactive oxygen species (ROS) attack and not by enzymatic regulation. Elevated DNA methylation level in highly hydroxymethylated spermatozoa detected in our study also suggests that 5hmC is not formed through 5mC oxidation by TETs, which is usually accompanied by a decrease in 5mC level. Occurrence of high sperm DNA fragmentation, which is mostly caused by ROS [37-39], in patients with elevated rates of 5hmC-positive spermatozoa is in favor of ROS-induced sperm hydroxylation as well.

The other issue to address is the stage of spermatogenesis at which a high 5hmC level is aberrantly induced in spermatozoa. We show that during spermatogenesis, hydroxymethylation is present in interphase spermatogonia Ad, is absent in all mitotic spermatogonial chromosomes, interphase spermatogonia Ap and B and spermatocyte meiotic chromosomes, and then appears again postmeiotically in a minor population of spermatids. This picture of 5hmC distribution does not vary significantly among azoospermic patients. Thus, hydroxymethylation pattern seems to undergo programmed genome-wide alterations in differentiating spermatogenic cells. During post-testicular sperm maturation, spermatozoa have strongly decreased amount of cytoplasm which makes their genetic and epigenetic patterns highly vulnerable to internal and external factors, including ROS [23, 40]. Therefore, we hypothesize that a high 5hmC level in individual ejaculated spermatozoa results from spontaneous post-testicular hydroxymethylation rather than programmed epigenetic changes.

Interestingly, in hierarchically organized human adult tissues, the lowest 5hmC levels are typical for stem/progenitor cells and the highest levels - for terminally differentiated cells [41]. The analysis of testicular cytogenetic and histological preparations in the present study and histological sections by Nettersheim et al. [42] shows that human testicular tissue is characterized by a different picture: by a high 5hmC level in stem cell spermatogonia Ad and a generally low 5hmC level at the further stages of differentiation. Spermatogenic cells have been supposed to lose 5hmC passively (in a replication-dependent manner) during mitotic/meiotic divisions [36, 42]. However, visualization of mitotic and meiotic chromosomes in our study shows a 5mC pattern similar to that reported previously in human lymphocytes and embryonic tissues [43-47] and no 5hmC (Figure 5). In neither of the >1200 chromosome spreads did we observe hydroxymethylation asymmetry in sister chromatids, which is a major sign of 5hmC passive loss, as shown in blastomeres of preimplantation embryos and in some somatic cells [48-51]. This suggests that 5hmC elimination from spermatogonia Ad most probably occurs before they enter mitosis to form spermatogonia Ap. It is noteworthy that oocyte meiotic chromosomes are characterized by high 5hmC and 5mC levels (Figure 8), thus, indicating strong sex-specific differences in gamete hydroxymethylation and methylation.

In conclusion, in human spermatogenesis, there seem to be two types of 5hmC pattern alterations: those strictly determined by germline-specific reprogramming in testicular tissue and those sporadically induced by endo- and exogenous factors in individual post-testicular spermatozoa. The immunocytochemically detected percentage of these highly hydroxymethylated spermatozoa in ejaculate is associated with fertility status and sperm parameters and, thus, can be suggested as a novel indicator of semen quality. The use of epigenetic analysis for categorization of infertile patients is supposed to significantly improve diagnostic approaches in the field of male reproduction [52, 53]. Moreover, understanding the mechanisms involved in epigenetic alterations opens the way for targeted treatment of pathological conditions [54, 55]. We believe that our findings are potentially significant for diagnosis and treatment of male infertility.

## MATERIALS AND METHODS

### Collection of testicular tissue samples, sperm and oocytes

Testicular tissue samples were obtained by open biopsy from 15 patients (24-54 yrs, mean 37.1±2.29) diagnosed with azoospermia. Of 15 patients, 7 had obstructive and 8 non-obstructive azoospermia. Immediately after sampling, the testicular tissue fragments were placed in Petri dish with Flushing medium (Origio, 10845060, Denmark), released from blood clots under the Leica M125 stereomicroscope, and stored no longer than 1 hr at room temperature before treatments for cytogenetic slide preparation.

Semen samples were obtained from 13 sperm donors (22-34 yrs, mean 28.6±1.1) and 37 patients from infertile couples (21-53 yrs, mean 34.9±1.05) by masturbation after 3-5 days of abstinence period. The sperm donors were physically and mentally healthy had normal karyotype and the following semen characteristics at the time of collection: volume ≥2 ml, concentration ≥64 millions/ml, progressive motility ≥49 %, normal morphology (according to [56]) ≥6 %, cryotolerance ≥50 %. The inclusion criteria for patients were infertility in a couple (failure to achieve pregnancy after a year of regular, unprotected intercourse) and normal karyotype, considering the possible effect of karyotype abnormalities on cell epigenetic status and DNA fragmentation [57-59]. The somatic karyotyping of the patients was performed by QFH-banding/actinomycin D (AcD) counterstaining of lymphocyte metaphase spreads according to the protocol repeatedly used in our center [60, 61]. Microdeletion analysis in Yq11 was performed and showed no alterations of AZF region in either patient. A conventional semen analysis (volume, pH, concentration, motility, vitality, and morphology) was performed according to WHO recommendations [62] and revealed normospermic (*n* = 21), asthenozoospermic (*n* = 5), oligoasthenozoospermic (*n* = 2) and teratozoospermic (*n* = 9) individuals among the patients. Spermiogram parameters of patients and sperm donors are summarized in Table 1.

**Table 1:** Semen parameters of analyzed sperm donors and patients from infertile couples.

Semen parameters	Sperm donors, *n*=13	Patients from infertile couples, *n*=37	*P*-values
Concentration (×10^6^/ml)	118.3 (64-191)	128.1 (2-340)	0.9434
Total motility (%)	86.15 (65-100)	87.92 (47-100)	0.5623
Progressive motility (%)	68.85 (49-90)	57 (17-88)	0.0487*
Non-progressive motility (%)	17.31 (3-38)	30.92 (1-83)	0.1472
Normal morphology (%)	12.69 (6-24)	5.946 (0-22)	0.0003*
Normal head (%)	13.62 (5-24)	4.235 (0-25)	<0.0001*
Abnormal morphology (%)	67.00 (28-94)	90.68 (73-100)	<0.0001*
Abnormal head (%)	74.08 (52-92)	85.97 (68-100)	0.0008*
Abnormal tail (%)	1.54 (0-10)	1.24 (0-11)	0.3751
Abnormal midpiece (%)	5.54 (0-22)	3.46 (0-12)	0.62

Semen parameters are shown as mean and range (minimum to maximum). *P*-values were calculated using the Mann-Whitney U-test. *P*-values showing statistically significant difference between the groups are marked by *.

Unfertilized oocytes were selected for the study as described previously [51]. After hormonal stimulation, oocytes were aspirated from patients’ follicles, rinsed in Flushing Medium (Origio, 10845060, Denmark) and incubated for 3 hrs in the ISM1 medium (Origio, 10500060, Denmark) at +37 °C in an atmosphere of 5 % CO_2_. Then, routinely prepared sperm was added for *in vitro* fertilization and after 20 hrs, the presence of pronuclei was examined. A total of 23 unfertilized oocytes were selected for the study.

The biological samples were obtained at the Department of Assisted Reproductive Technologies, D.O. Ott Research Institute of Obstetrics, Gynecology and Reproductology, in AVA-Peter Clinic and in Aimed Clinic (St. Petersburg, Russian Federation). The study was approved by the Ethics committee of D.O. Ott Research Institute of Obstetrics, Gynecology and Reproductology. All the samples were enrolled in the study after patients signed the informed consent. The study was performed in accordance with the Declaration of Helsinki.

### Cytogenetic slide preparation

Chromosomes and nuclei from the testicular tissue samples were fixed on glass slides by the direct technique (without *in vitro* culture) [63]. Shortly, testicular tissue fragments were incubated in hypotonic solution (0.9 % sodium citrate) with colchicines (2.5 μg/ml) at room temperature for 60-80 min and then in fixative (methanol : glacial acetic acid, 3:1) at +4 °C for 90 min. Then they were macerated in drops of 60 % acetic acid on glass slides. After saturation with cells, drops of 60 % acetic acid were spread on the slides, fixed with 2-3 drops of fixative (methanol : glacial acetic acid, 3:1) and air-dried.

Sperm slides were prepared from 1 ml aliquots of native semen samples as described previously [51]. The semen samples were treated with hypotonic solution (0.9% sodium citrate) at room temperature for 20 min. Then, they were incubated at +4 °C in freshly prepared fixative (methanol : glacial acetic acid, 3:1), which was changed at least twice during a 60 min fixation period. The suspension was concentrated by centrifugation and dropped onto slides. The slides were air dried at room temperature.

Oocyte chromosome preparations were made according to the fixation protocol for single cells [64]. Unfertilized oocytes were treated with 0.1% colchicines for 6-12 hrs prior fixation, incubated for 10-30 s in a drop of 0.9% sodium citrate supplemented with 1% protease, washed in 0.9% sodium citrate solution, and fixed on the slides with freshly prepared cold fixative (methanol : glacial acetic acid, 3:1).

### Tissue slide preparation

Tubular cross section slides were prepared from the testicular tissue fragments which had not been used for cytogenetic preparations and had been stored in fixative (methanol : glacial acetic acid, 3:1). Such tissue fragments were available from 5 patients. The tissue fragments were transferred to 10 % formalin and incubated for 24 hrs. Then, the tissue fragments were washed in flowing water for 30 min and processed for paraffin embedding in Logos J (Milestone, Italy) as recommended by the manufacturer. 2.5 µm thick tissue sections were cut on a rotary microtome HM 340E (Thermo Scientific, Germany) and placed on Superfrost Plus glass slides. After incubating the slides at +37 °С overnight, heat-induced epitope retrieval was performed in the PT-Module (Thermo Scientific, Germany) in a recommended buffer (pH = 6) (Thermo Scientific, Germany) at +98 °С for 30 min. Then, buffer with the slides was cooled down to +65 °С, and the slides were transferred to room temperature 1хPBS for subsequent immunostaining.

### FISH

To determine nuclei ploidy, FISH was performed on the testicular cytogenetic preparations according to standard procedures with minor modifications [65]. After aging at +55 °C overnight, the slides were dehydrated in ethanol series (70 %, 80 % and 95 %), washed in 4xSSC at +37 °C for 3 min, incubated in 0.01 % pepsin solution at +37 °C for 25 min and washed again in 4xSSC at +37 °C for 3 min. Then, the slides were fixed in 2.5% paraformaldehyde 2xSSC solution at room temperature for 10 min, washed in two changes of fresh 4xSSC at +37 °C for 3 min, rinsed in distilled water and dehydrated in ethanol series. 10 μl of hybridization mixture was then applied to each slide, covered with 22x22 mm coverslip and sealed with rubber cement. Two DNA-probes were used: Vysis CEP 3 (D3Z1) SpectrumOrange Probe (3p11.1-q11.1 Alpha Satellite DNA) and Vysis CEP 10 SpectrumAqua Probe (10p11.1-q11.1) (Abbott Molecular, USA). The use of two DNA-probes instead of one helps to avoid misinterpretation of the hybridization picture, when a diploid nucleus with an occasional loss of one chromosome can be classified as a haploid one. The slides with sample and probe mixture were denatured for 10 min at +78 °C and then hybridized for at least 12 hrs at +37 °C in Thermobrite. Then, rubber cement was carefully removed and the slides were washed in 4xSSC, supplemented with Tween 20 (0.5 %) at +37 °C until coverslips off. The slides were washed in two changes of 4xSSC at +37 °C for 3 min, rinsed in distilled water, dehydrated in ethanol series and air-dried.

### Immunodetection of 5hmC and 5mC

The immunodetection of 5hmC and 5mC was performed using primary antibodies against 5hmC (rabbit polyclonal, Active Motif, 39769, USA) and 5mC (mouse monoclonal, Millipore, clone 33D3, MABE146, USA) and secondary goat anti-rabbit Alexa Fluor 488 (Life Technologies, A-11008) and goat anti-mouse Alexa Fluor 594 (Life Technologies, A-11005, USA) / Alexa Fluor 555 (Life Technologies, A-21424, USA) antibodies according to the protocol used previously [53]. The preparations were denatured in 2 M HCl for 20-30 min at room temperature, washed in ice-cold PBS and distilled water and incubated with blocking solution (1 % BSA, 0.1 % Tween 20 in PBS) for 30-40 min at +37 °C in a humidified chamber. Then, the mixture of primary antibodies diluted in blocking solution (1:500) was applied to the slides for 90 min at room temperature in the humidified chamber. On the negative control slides, the primary antibody step was omitted. The slides were washed thrice with PBS for 15 min each, supplemented with 0.5 % Tween 20 at +43 °C in a shaking bath and the mixture of secondary antibodies, diluted in blocking solution (1:200) was applied to the slides for 60 min at +37 °C in the humidified chamber. The slides were then washed thrice with PBS for 15 min each supplemented with 0.5 % Tween 20 at +43 °C in the shaking bath, rinsed in PBS and distilled water, dehydrated in the ethanol series (70, 80, and 96 %), and mounted in DAPI-containing Vectashield antifade (Vector Laboratories, H-1200, USA).

For simultaneous analysis of 5hmC patterns and nuclei ploidy on the cytogenetic slides, FISH was performed prior to the immunodetection of 5hmC. In this case, the step of DNA denaturation was omitted in the immunodetection protocol, as denaturation was performed earlier during the FISH procedures. The step of DNA denaturation in the immunodetection protocol was also omitted for the histological slide preparations.

DNA hydroxymethylation and methylation patterns were first analyzed qualitatively based on visual assessment of 5hmC or 5mC presence/absence in testicular cells and ejaculated spermatozoa. In testicular cells, the 5hmC and 5mC patterns were analyzed in 5000 nuclei and in 15-25 chromosome spreads from mitotic spermatogonia at metaphase stage, meiotic spermatocyte at prophase I (pachytene, diplotene, and diakinesis) and metaphase II stages from each of 15 azoospermic patients. Diploid nucleus was considered to be from spermatogonium Ad, if a large central vacuole-like cavity was detected [33]. All haploid nuclei were considered to be from postmeiotic spermatogenic cells. In ejaculates, 5hmC and 5mC patterns were analyzed in 5000 spermatozoa from each of 37 patients and 13 sperm donors. After visual analysis, the measurements of anti-5hmC and anti-5mC fluorescence intensity were performed on the representative digital images of 300 5hmC-positive and 300 5hmC-negative spermatozoa from 3 individuals. The intensity of fluorescence was calculated in arbitrary units (a.u.) for each spermatozoa using Image J 1.48v software.

### TUNEL

To assess sperm DNA integrity, TUNEL was performed on the sperm slides according to the protocol reported previously [66], with modifications. The preparations were washed twice in 1xPBS for 5 min each and then additionally fixed in 4 % paraformaldehyde for 10 min at room temperature. The preparations were permeabilized by treatment with 0.1 % Triton X-100 in 0.1 % sodium citrate for 15 min at +4 °C. Then, the slides were washed twice in 1xPBS for 5 min each at room temperature and a reagent mixture (4.5 µl of nucleotide mixture in reaction buffer + 0.5 µl of enzyme (terminal deoxynucleotidyl transferase from calf thymus, recombinant in E. coli, in storage buffer, Roche, Germany) was added. After incubation for 1 h at +37 °С in the humidified chamber, the slides were washed thrice in 1xPBS for 5 min each at room temperature, rinsed with distilled water, dehydrated in the ethanol series (70, 80, and 96 %), and mounted in DAPI-containing Vectashield antifade (Vector Laboratories, USA). The presence of single- and double-strand breaks in DNA was registered by a microscopically detected fluorescent signal in the sperm head. To calculate the percentage of sperm with fragmented DNA, a total of 2000 sperm heads were analyzed in each sample.

### Image acquisition

Fluorescence images were acquired using a Leica DMLS microscope with a Leica DFC320 camera and Leica DFC Twain software (for QFH/AcD banded chromosomes), as well as a Leica DM 2500 microscope with SOR, SGR, SRE and A filtercubes, a Leica DFC345 FX camera and Leica Application SuiteV.3.8.0 software (for phase contrast, FISH, immunostained, and DAPI-stained preparations). Time of exposition for images was up to 5.5 s for 5hmC (green), up to 2 s for 5mC (orange/red). Individual images were processed in Adobe Photoshop CS3 with “crop”, “brightness/contrast” and “sharpen” instruments applied to the whole image.

### Statistical analysis

The statistical analysis was performed using GraphPad Prism, Version 6.01 and Microsoft Office Excel 2003 SP2. Values are shown as mean and range (minimum to maximum) for semen parameters, and as mean and standard deviation (SD) for testicular cell nuclei counts. Data distribution was evaluated by the D’Agostino-Pearson test. Non-parametric variables were compared using the Mann-Whitney U-test. The correlation coefficients were calculated using the nonparametric Spearman test. The proportions of hydroxymethylated and non-hydroxymethylated diploid and haploid nuclei from testicular samples of patients with obstructive and non-obstructive azoospermia were compared using Fisher’s z-test. The α-level was set at 0.05.
